# Improvements of predictive power of B-type natriuretic peptide on admission by mathematically estimating its discharge levels in hospitalised patients with acute heart failure

**DOI:** 10.1136/openhrt-2021-001603

**Published:** 2021-05-17

**Authors:** Eiji Anegawa, Hiroyuki Takahama, Kunihiro Nishimura, Daisuke Onozuka, Yuki Irie, Kenji Moriuchi, Masashi Amano, Atsushi Okada, Makoto Amaki, Hideaki Kanzaki, Teruo Noguchi, Kengo Kusano, Satoshi Yasuda, Chisato Izumi

**Affiliations:** 1 Department of Cardiovascular Medicine, National Cerebral and Cardiovascular Center, Suita, Japan; 2 Department of Preventive Medicine and Epidemiology, National Cerebral and Cardiovascular Center, Suita, Japan

**Keywords:** heart failure, biomarkers, heart failure, diastolic, heart failure, systolic Pl

## Abstract

**Backgrounds:**

Earlier studies showed that in patients with heart failure (HF), circulating levels of B-type natriuretic peptide (BNP) at hospital discharge (BNP_dis_) are more predictive of prognosis than BNP levels on admission (BNP_ad_). However, the mechanism underlying that difference has not been fully elucidated. We examined the association between confounding factors during hospitalisation and BNP_dis_ in patients with HF.

**Methods:**

We identified patients admitted to our hospital for HF (BNP_ad_ ≥100 pg/mL). Estimated left ventricular end-diastolic pressure (eLVEDP) was calculated using echocardiographic data. To identify the factors associated with the relation between BNP_ad_ and BNP_dis_, we performed a stepwise regression analysis of retrospective data. To validate that analysis, we performed a prospective study.

**Results:**

Through stepwise regression of the patient data (n=688, New York Heart Association 3–4, 88%), we found age, blood urea nitrogen and eLVEDP to be significantly (p<0.05) associated with BNP_dis_. Through multivariate analysis after accounting for these factors, we created a formula for predicting BNP levels at discharge (*predicted*-BNP_dis_) from BNP_ad_ and other parameters measured at admission (p<0.05). By statistically adjusting for these factors, the prognostic power of BNP_ad_ was significantly improved (p<0.001). The prospective study also confirmed the strong correlation between *predicted*-BNP_dis_ and BNP_dis_ (n=104, r=0.625, p<0.05).

**Conclusion:**

This study showed that statistically accounting for confounding factors affecting BNP levels improves the predictive power of BNP levels measured at the time of hospital admission, suggesting that these confounding factors are associated with lowering predictive power of BNP on admission.

**Trial registration number:**

UMIN 000034409, 00035428.

**Key questions**

**What is already known about this subject?**

- Predictive power of B-type natriuretic peptide (BNP) levels at hospital admission (BNP_ad_) is inferior to that of BNP levels at discharge (BNP_dis_) in patients with acute heart failure (HF). However, the mechanism underlying these differences has not been fully elucidated.

**What does this study add?**

- Through multivariate analysis after accounting for known confounding factors related to circulating BNP levels, we created a formula for predicting BNP levels at discharge (*predicted*-BNP_dis_) from BNP_ad_ and other parameters measured at admission.
- This study showed that statistically accounting for confounding factors affecting BNP levels improves the predictive power of BNP levels measured at the time of hospital admission.

**How might this impact on clinical practice?**

- This *predicted*-BNP_dis_ has superior predictive power for clinical outcomes to BNP_ad_. This may contribute to the risk stratification of acute HF and primary intensive care management in acute vulnerable phases of HF.

## Introduction

B-type natriuretic peptide (BNP) is widely used as a predictive biomarker in patients with heart failure (HF).^1^ However, earlier studies showed that in patients with acute HF, the predictiveness of BNP levels measured at hospital admission (BNP_ad_) for clinical outcomes is inferior to BNP levels measured at discharge (BNP_dis_).^2 3^ Although the reason for the insufficient predictive power of BNP_ad_ compared with BNP_dis_ in these patients has not been fully elucidated, several confounding factors are well known to influence circulating BNP levels (eg, left ventricular end-diastolic pressure (LVEDP) and renal function).^4–7^ However, how these parameters influence the lower predictability of BNP_ad_ than that of BNP_dis_ remains uncertain.

Given the importance of clinical risk stratification for hospitalised patients with heterogeneous clinical syndromes, this study aimed to identify the confounding factors affecting BNP_ad_ that are associated with BNP_dis_. We also tested the hypothesis: whether statistical adjustments of these confounding factors are related to predictive power of BNP_ad_ improvements in patients with acute HF.

## Methods

### Study design

This was a cross-sectional study of patients with HF admitted to the National Cerebral and Cardiovascular Center of Japan.

### Study population

#### Retrospective study

Included in the retrospective study were 688 patients hospitalised for HF between January 2013 and March 2016 (BNP on admission: ≥100 pg/mL). We excluded patients who did not undergo a blood test and echocardiography, who underwent implantation of a left ventricular (LV) assist device (n=3), or who died in the hospital (n=24) during the corresponding hospitalisation. We also excluded patients who underwent mitral valve surgery (n=85) due to its influence on transmitral flow and septal mitral annular velocity.

Diagnosis of HF was based on the Framingham criteria.^8^ Whether or not a HF episode met the Framingham criteria was determined by each attending physician and an investigator (H Takahama) via medical record review. We excluded patients who did not meet the criteria, as judged by the investigator and the attending physician for each patient. According to the guidelines of the Japanese Circulation Society,^9^ we defined the cut-off value of plasma BNP level for diagnosis of HF as 100 pg/mL.

#### Prospective study

We prospectively collected data from 104 patients between January and June in 2019 based on the same criteria used for the retrospective study.

#### Echocardiography

Through medical chart review, we retrospectively reviewed the echocardiography data collected during the hospitalisation. LV dimensions were measured according to the American Society of Echocardiography guidelines.^10^ LV ejection fraction (EF) was measured using the modified Simpson method or the semiquantitative two-dimensional visual estimate method, as described previously.^11^ Transmitral inflow was measured with pulsed-wave Doppler using standard methods as described previously.^12^ The septal mitral annular early diastolic velocity (e′) was determined with spectral tissue Doppler imaging. LVEDP was calculated as 11.96+0.596 × early diastolic transmitral flow velocity (E)/e′, as previously reported.^13^

#### Measurement of plasma BNP concentration

All biochemical analyses were performed as routine clinical examinations. BNP were measured by human brain natriuretic peptide kit (TOSOH corporation, Tokyo, Japan).

#### Clinical outcomes

After the admission date, we investigated through medical chart review or a letter all causes of death and rehospitalisation for HF. Combined clinical events were defined as all-cause death or rehospitalisation for HF.

#### Ethics

The study was designed to be carried out without obtaining individual informed consent according to the ‘opt-out’ principle. Instead, we publicised a summary of the study protocol with the contact information for our office on the institution website, which provided patients with the ability to refuse enrolment to the study. This study protocol was also registered in the Japanese University Hospital Medical Information Network Clinical Trials Registration.

### Statistical analyses

Results are expressed as the median and IQR. Fisher’s exact test or the χ^2^ test was used to compare categorical variables, as appropriate. With regard to baseline patient characteristics, Wilcoxon’s rank-sum test was used for comparison of continuous variables between two groups. HRs with 95% CIs and probability (p) values determined using the likelihood ratio test are presented. The area under the receiver operating characteristics (ROC) curve (AUC) and C-statistics were also calculated. AUCs were compared using an algorithm developed by DeLong *et al*.^14^ Pairwise comparisons of the areas under multiple ROC curves were made using the roccomp command in Stata. Multivariate analysis/regression was used to test multiple covariates. All tests were two tailed, and values of p<0.05 were considered significant. All statistical analyses were performed using JMP V.9 statistical analysis software (SAS Institute Japan, Inc, Tokyo, Japan) and Stata V.15 (Stata Corporation LLC, College Station, Texas, USA).

## Results

### Retrospective data analysis

Using the inclusion and exclusion criteria described in the Methods section, we identified 688 patients with HF from our database. The patient characteristics on admission were as follows (table 1): New York Heart Association class III and IV on admission, 43% and 45%, respectively; median LVEF: 35% (IQR: 24%–55%); median LV end-diastolic diameter (LVEDD), 56 mm (IQR: 48–64 mm); and median plasma BNP levels, 671 pg/mL (IQR: 370–1170 pg/mL). Median hospitalisation length was 19.5 days (IQR: 14–27 days). The patients treated without beta-blockers were often observed in those with valvular regurgitation and with HF with preserved EF. Based on earlier studies, we selected the following clinical parameters known to influence circulating BNP levels for analysis: age, sex,^4^ LVEDP,^5 15^ blood pressure,^16^ heart rate,^7^ body mass index (BMI),^6^ LVEF,^17 18^ end-diastolic volume,^17^ LVEDD,^18^ cholesterol levels,^19^ anaemia,^20^ renal function,^7 20^ atrial fibrillation^21^ and diabetes mellitus.^22^ The results of univariate analyses of the association between BNP_dis_ and the selected parameters are shown in table 2. Age, BMI, systolic blood pressure, diastolic blood pressure, LVEDD, LVEF, estimated LVEDP (eLVEDP), estimated glomerular filtration, blood urea nitrogen (BUN), haemoglobin, hematocrit, total cholesterol and low-density lipoprotein cholesterol on admission were all associated with BNP_dis_ after adjusting for BNP_ad_. Stepwise analysis identified the following parameters as significantly associated with BNP_dis_ after accounting for BNP_ad_ levels: age, systolic blood pressure, LVEF, eLVEDP and BUN levels (table 3). Subsequent multivariate analysis revealed the parameter estimates of the above factors to be: 3.81 (age), −1.09 (systolic blood pressure), −0.87 (LVEF), 6.72 (eLVEDP), 3.48 (BUN) and 0.21 (BNP_ad_). Using those data, we developed the following formula to predict BNP_dis_ from BNP_ad_: the ‘*predicted*-BNP_dis_’=3.81 × age – 1.09 × systolic blood pressure – 0.87×LVEF + 6.72 × eLVEDP + 3.48 × BUN + 0.21 × BNP_ad_ – 184.5. Next, the analysis of association non-pharmacological intervention on the relation between the parameters used for estimation of the *predicted*-BNP_dis_ and BNP_dis_ were performed because the therapeutic intervention might influence the discharge levels of BNP. As shown in online supplemental table 1, no statistical significance was found. In addition, there was significant differences in both BNP_ad_ and BNP_dis_ in patients with initial admission and rehospitalisation for HF. As shown in online supplemental table 2, both BNP levels were higher in patients with readmission than those in initial admission. The correlation of the *predicted*-BNP_dis_ with BNP_dis_ was similar between the patients with initial admission and readmission (p<0.05).

10.1136/openhrt-2021-001603.supp1 Supplementary data

10.1136/openhrt-2021-001603.supp2 Supplementary data

**Table 1:** Baseline patient characteristics

	Overall patients
Patients number	688
Age (years)	78 (69–84)
Gender (male)	429 (62)
BMI (kg/m²)	22.5 (20.2–25.4)
NYHA class	
Class III	296 (43)
Class IV	311 (45)
Aetiology	
Ischaemic	232 (34)
Non-ischaemic	172 (25)
Valvular	98 (14)
Hypertensive	149 (22)
Others	37 (5)
**History**	
Hypertension	490 (71)
Atrial fibrillation	345 (50)
Diabetes mellitus	250 (36)
**Vital signs and others on admission**	
Systolic blood pressure (mm Hg)	120 (105–138)
Diastolic blood pressure (mm Hg)	68 (58–81)
Heart rate (bpm)	77 (66–92)
**Echocardiography**	
LVEDD (mm)	56 (48–64)
LVESD (mm)	44 (34–54)
LVEF (%)	35 (24–55)
E/e′	14.4 (10.6–19.4)
eLVEDP (mm Hg)	20.5 (18.3–23.5)
**Laboratory data**	
BNP_ad_ (pg/mL)	671 (370–1170)
BNP_dis_ (pg/mL)	280 (153–468)
eGFR (mL/min/1.73 m^2^)	46 (30–60)
BUN (mg/dL)	24 (18–34)
Hb (g/dL)	12.2 (10.7–13.4)
Hct (%)	37 (33–41)
CRP (mg/dL)	0.35 (0.12–1.20)
WCC (×10^3^/uL)	6.4 (5.1–8.0)
T-Cho (mg/dL)	154 (130–178)
HDL-C (mg/dL)	42 (34–51)
LDL-C (mg/dL)	88 (70–110)
**Medications**	
**On admission**	
ACEi or ARB	283 (41)
Beta-blockers	401 (58)
Aldosterone antagonists	186 (27)
Loop diuretics	418 (61)
Dobtamin	68 (10)
**At discharge**	
ACEi or ARB	516 (75)
Beta-blockers	539 (78)
Aldosterone antagonists	297 (43)
Loop diuretics	585 (85)
Median observation period (days)	623 (188–730)

Values are the median (IQR) and patients number, n (%).

ACEi, ACE inhibitor; ARB, angiotensin II receptor blocker; BMI, body mass index; BNP_ad_, circulating B-type natriuretic peptide levels on admission; BNP_dis_, circulating B-type natriuretic peptide levels at hospital discharge; BUN, blood urea nitrogen; CRP, C reactive protein; eGFR, estimated glomerular filtration rate; eLVEDP, estimated left ventricular end-diastolic pressure; Hb, haemoglobin; Hct, haematocrit; HDL-C, high-density lipoprotein cholesterol; LDL-C, low-density lipoprotein cholesterol; LVEDD, left ventricular end-diastolic diameter; LVEF, left ventricular ejection fraction; LVESD, left ventricular end-systolic diameter; NYHA, New York Heart Association; T-cho, total cholesterol; WCC, white cell count.

**Table 2:** Association of discharge levels of BNP with clinical parameters on admission (univariate analysis)

Variables	*r*	P value
Age	0.1550	<0.0001
Gender	n/a	0.3940
BMI (kg/m²)	−0.2167	<0.0001
Atrial fibrillation	n/a	0.1084
Diabetes mellitus	n/a	0.9110
Systolic blood pressure (mm Hg)	−0.1109	0.0036
Diastolic blood pressure (mm Hg)	−0.0875	0.0220
Heart rate (bpm)	−0.0657	0.0854
LVEDD (mm)	0.1084	0.0045
LVEF (%)	−0.1822	<0.0001
eLVEDP (mm Hg)	0.1460	0.0001
BNP_ad_ (pg/mL)	0.5779	<0.0001
eGFR (mL/min/1.73 m^2^)	−0.3153	<0.0001
BUN (mg/dL)	0.3438	<0.0001
Hb (g/dL)	−0.1635	<0.0001
Hct (%)	−0.1529	<0.0001
T-Cho (mg/dL)	−0.1312	0.0006
HDL-C (mg/dL)	−0.0362	0.3476
LDL-C (mg/dL)	−0.0793	0.0388

Abbreviations are shown in table 1.

**Table 3:** Stepwise regression analysis for association of BNP_dis_ with variables on admission

Variables	P value
Age	0.0004
BMI (kg/m²)	0.7260
Systolic blood pressure (mm Hg)	0.0074
Diastolic blood pressure (mm Hg)	0.4822
LVEDD (mm)	0.1740
LVEF (%)	0.0157
eLVEDP (mm Hg)	0.0066
eGFR (mL/min/1.73 m^2^)	0.6155
BUN (mg/dL)	<0.0001
Hb (g/dLl)	0.0656
Hct (%)	0.4669
T-Cho (mg/dL)	0.6299
LDL-C (mg/dL)	0.7344

Stepwise regression analysis for association of BNP_dis_ with variables on admission after accounting for BNP_ad_. Abbreviations are shown in table 1.

**Figure 1:**
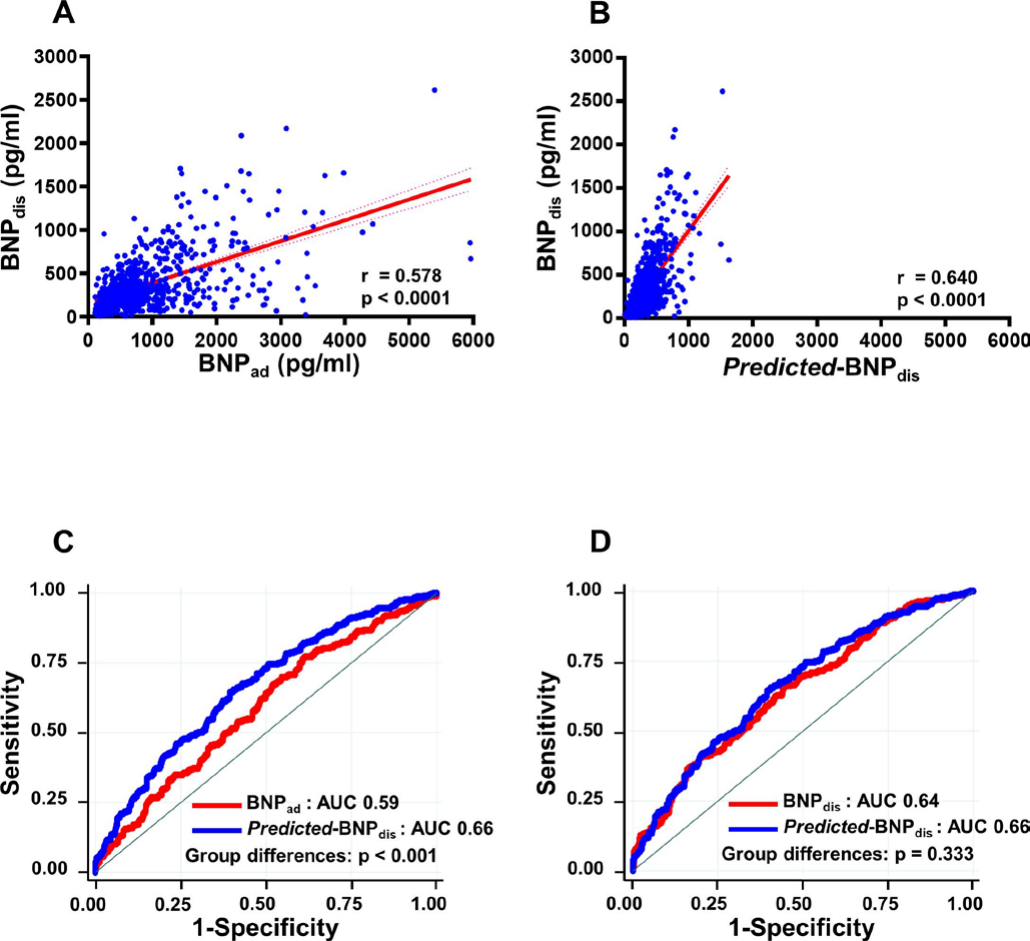
Association between BNP levels at hospital discharge and levels on admission or predicted BNP levels at discharge (*predicted*-BNP_dis_) and their prognostic power. (A) Correlation between circulating BNP levels at hospital discharge (BNP_dis_) and BNP on admission (BNP_ad_). (B) Correlation between BNP_dis_ and predicted BNP levels at discharge (*predicted*-BNP_dis_).(C) Area under the receiver operating characteristics curve (AUC) analysis of the occurrence of the combined clinical events. AUC for the predicted BNP levels at discharge (*predicted*-BNP_dis_: blue) was superior to BNP levels on admission (BNP_ad_: red) (p<0.001). (D) There was no significant difference in the AUC for the *predicted*-BNP_dis_ (blue) and BNP levels at discharge (BNP_dis_: red). BNP_ad_, B-type natriuretic peptide on hospital admission; BNP_dis_, B-type natriuretic peptide at discharge.

During the follow-up period (median: 623 days, IQR: 188–730 days), combined clinical events occurred in 295 (43%) patients: all causes of death (n=68, 10%) and rehospitalisation for HF (n=227, 33%). Figure 1 shows the correlation between BNP_dis_ and BNP_ad_ (figure 1A) and between BNP_dis_ and *predicted*-BNP_dis_ (figure 1B). However, AUC analysis showed that *predicted*-BNP_dis_ was significantly more predictive of outcome than BNP_ad_ (p<0.001) and was comparable with the predictiveness of BNP_dis_ (figure 1C, D).

### Prospective data analysis

**Figure 2:**
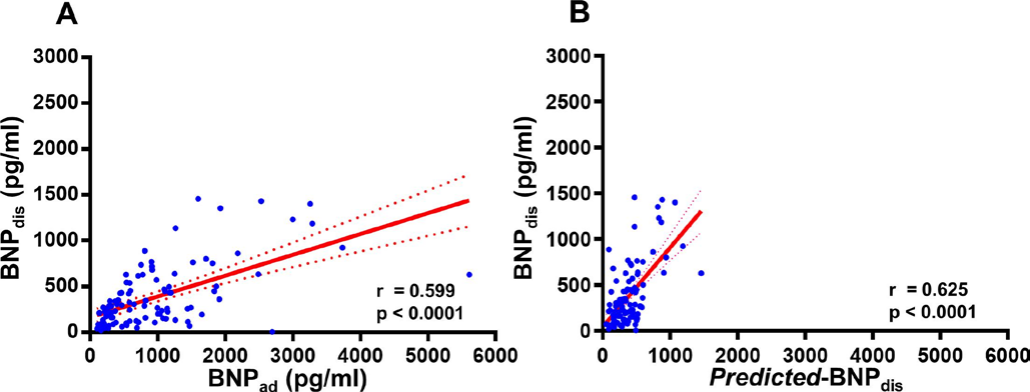
Association between BNP levels at hospital discharge and levels on admission or predicted BNP levels at discharge (*predicted*-BNP_dis_) in a prospective study. (A) Correlation between circulating BNP levels at hospital discharge (BNP_dis_) and BNP levels on admission (BNP_ad_). (B) Correlation between BNP_dis_ and predicted BNP levels at discharge (*predicted*-BNP_dis_). Note the similarity between the results of the prospective and retrospective studies. BNP_ad_, B-type natriuretic peptide on hospital admission; BNP_dis_, B-type natriuretic peptide at discharge.

The prospective study revealed similar relationships between BNP_ad_ or *predicted*-BNP_dis_ and BNP_dis_ (figure 2A, B).

## Discussion

Circulating BNP levels are affected by a number of confounding factors during the acute phase of HF and are associated with fluctuations in measured BNP levels during the initial few days after hospital admission or intensive treatments.^23 24^ These fluctuations are mainly due to changes in ventricular preload and the systemic fluid overload state. Our retrospective study confirmed known confounding factors affecting BNP levels and showed that these factors statistically were associated with lowering the predictive power of BNP_ad_ levels for patient outcomes. This study also showed that after statistically accounting for these confounding factors, the predictive power of the resultant BNP value (*predicted*-BNP_dis_) did not significantly differ from that of BNP_dis_. Moreover, the finding of the correlation of *predicted*-BNP_dis_ with BNP_dis_ was validated by obtaining similar results in a prospective study.

Several earlier studies reported on the factors influencing circulating BNP levels. For example, Iwanaga *et al*^5^ measured LVEDP using a LV catheter system and clearly demonstrated that LV wall stress strongly correlated with plasma BNP levels. In addition, factors such as increased cardiac preload likely due to excess body fluid, which may stretch ventricular cardiomyocytes, sharply increases circulating BNP levels in patients with HF. By the time BNP_dis_ levels are measured, however, the patient has reached an appropriate volume state through removal of the excess body fluid. Consequently, BNP_dis_ levels may more closely reflect myocardial quality per se or a ‘true ventricular trait’. This may explain why BNP_ad_ levels measured during the acute phase of HF have less predictive power than BNP_dis_ levels. In the present study, we observed that after accounting for several factors, including LVEDP and LVEF, as well as blood pressure and renal function, the predicted BNP levels, which we termed *predicted*-BNP_dis_, strongly correlated with BNP_dis_ levels and were equally predictive of patient outcome.

Thus, by determining the impact of factors responsible for the difference in predictive power between BNP_ad_ and BNP_dis_, we were able to shed light on the relationship between these factors and BNP_dis_. These findings may further our understanding of BNP levels, which are influenced by various factors in patients with acute HF.

### Limitation

The present study has several limitations. First, this was a single-centre investigation with a limited number of patients. Nevertheless, we were able to confirm a formula that predicts BNP levels at discharge from clinical parameters at the time of admission. Second, several patients did not undergo a blood test for BNP and E/e′; we excluded these patients from our analysis. Next, it is widely known that the therapeutic intervention might influence the discharge levels of BNP. These therapeutic interventions might also influence the relationship between the clinical parameters on admission with the BNP_dis_, although no statistical association were found in online supplemental table 1. Furthermore, this study enrolled the patients between 2013 and 2016, and at that time, sacubtrilvarsartan was not approved in Japan, which is known to influence circulating BNP levels. Further investigation will be necessary to confirm the effects of sacubtrilvarsartan on the predicted BNP levels at discharge. The association of the *predicted*-BNP_dis_ with BNP_dis_ is statistically significant, but the degree of correlation was modest; we could not exclude the possibility that the other unknown factor or therapeutic effects, which are not included for the estimation of BNP_dis_ in this study, might be also associated with the regulation of BNP. Taken together, this study was not designed to investigate the effects of the prospectively controlled pharmacological or non-pharamacological intervention on BNP_dis_. Further prospective study will be necessary to address these problems.

In addition, there was no discharge criteria for the research in this study, and in general, discharges were determined by the attending physician. Although the discharge levels are determined by clinical findings including BNP levels in stable phases of HF, the variation of HF severity at discharge exists among the attending physicians, which might create the further variation of the discharge levels of BNP.

## Conclusion

We have shown the confounding factors affecting measured BNP levels and demonstrated BNP prediction at discharge in hospitalised patients with HF. This predicted BNP values have superior predictive power for clinical outcomes to raw BNP values on admission. This may contribute to the risk stratification of acute HF and primary intensive care management in acute vulnerable phases of HF.

## Data Availability

No data are available.
