# Supplementary material for: Improvements of predictive power of B-type natriuretic peptide on admission by mathematically estimating its discharge levels in hospitalised patients with acute heart failure
Source: Open Heart. 2021 May 17;8(1):e001603. doi: 10.1136/openhrt-2021-001603 (PMC8130754; doi:10.1136/openhrt-2021-001603)
Supplement: Supplementary data [file openhrt-2021-001603supp001.pdf]

Supplemental Table 1 The association of non-pharmacological intervention with the relationship between the clinical parameters used for the estimation of the *predicted*-BNP_{dis} and BNP_{dis}.

Variables	p Value
Cardiac resynchronization therapy	0.1159
Percutaneous coronary intervention	0.1559
Catheter ablation	0.9407
Valve surgery	0.7322
Non-pharmacological intervention (any above)	0.0814
