# Supplementary material for: Improvements of predictive power of B-type natriuretic peptide on admission by mathematically estimating its discharge levels in hospitalised patients with acute heart failure
Source: Open Heart. 2021 May 17;8(1):e001603. doi: 10.1136/openhrt-2021-001603 (PMC8130754; doi:10.1136/openhrt-2021-001603)
Supplement: Supplementary data [file openhrt-2021-001603supp002.pdf]

Supplemental Table 2 Comparison of BNP levels in patients with initial admission and readmission for heart failure

	Overall	Heart failure		
		initial admission	readmission	p Value
Patients number	688	396	292	
BNP_{ad} (pg/ml)	671 (370, 1170)	592 (347, 1048)	740 (443, 1362)	0.0003
BNP_{dis} (pg/ml)	280 (153, 468)	240 (133, 376)	324 (194, 551)	< 0.0001
